# Supplementary figures and images for: The repositioning of epigenetic probes/inhibitors identifies new anti-schistosomal lead compounds and chemotherapeutic targets
Source: PLoS Negl Trop Dis. 2019 Nov 15;13(11):e0007693. doi: 10.1371/journal.pntd.0007693 (PMC6881072; doi:10.1371/journal.pntd.0007693)

A)

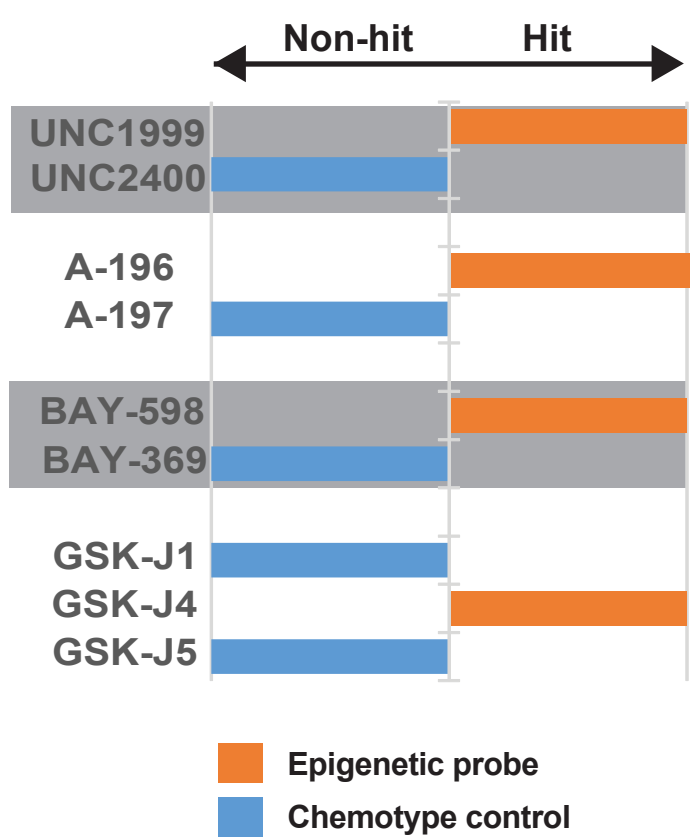

B)

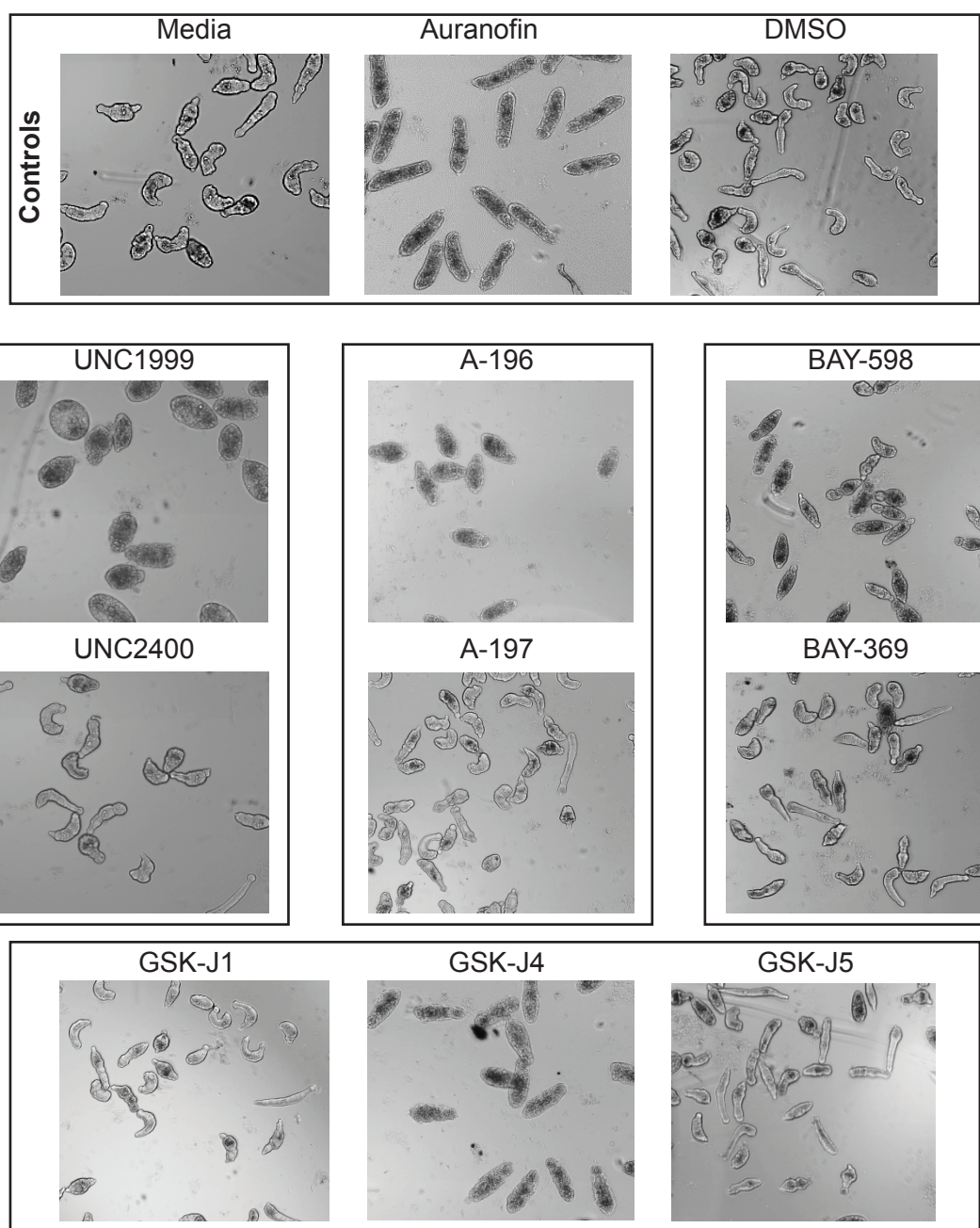

Supplement: S1 Fig — Chemotype-matched control compounds of EPs UNC1999 (UNC2400), A-196 (A-197), BAY-598 (BAY-369) and GSK-J4 (GSK-J1 and GSKJ5) as well as Auranofin were screened against S. mansoni schistosomula at 10 μM for 72 h using the Roboworm platform as described in the Methods. (A) Compounds were listed as a hit if they fell within both phenotype and motility cut-off thresholds (-0.15 and -0.35, respectively) [50]. (B) Representative images of schistosomula phenotypes induced by co-cultivation with these EPs and chemotype matched controls, compared to schistosomula co-cultivated with media only, DMSO (0.625%) and Auranofin. (PDF) [file pntd.0007693.s002.pdf]

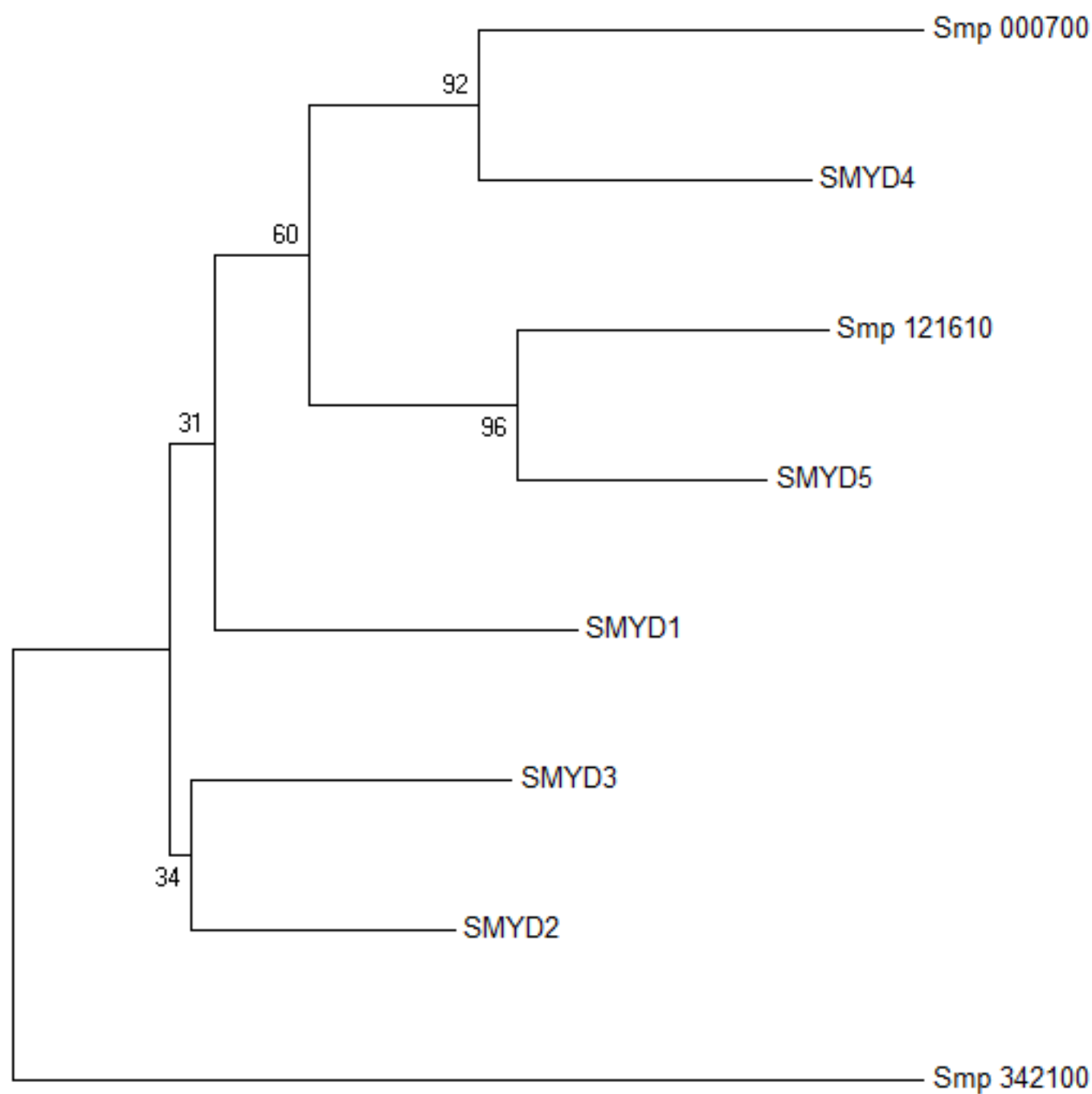

0.50

Supplement: S2 Fig — The phylogeny outlined in the tree is derived from multiple sequence alignment of the SET domain of 3 SmSMYDs (Smp_000700, Smp_121610 and Smp_342100) and 5 HsSMYDs (SMYD1-5). The consensus tree is constructed in MEGA using the neighbor joining method. An unrooted dendogram represents the bootstrap analysis of the HsSMYD and SmSMYD members accomplished using 1000 iterations. The taxa name (sequence name) is reported at the tip of each branch and the bootstrap value (supportive value) is indicated for each node. The branch length is proportional to the distance calculated between the various SMYD family members with the scale reported as reference at the bottom of the dendrogram. (PDF) [file pntd.0007693.s003.pdf]

A)

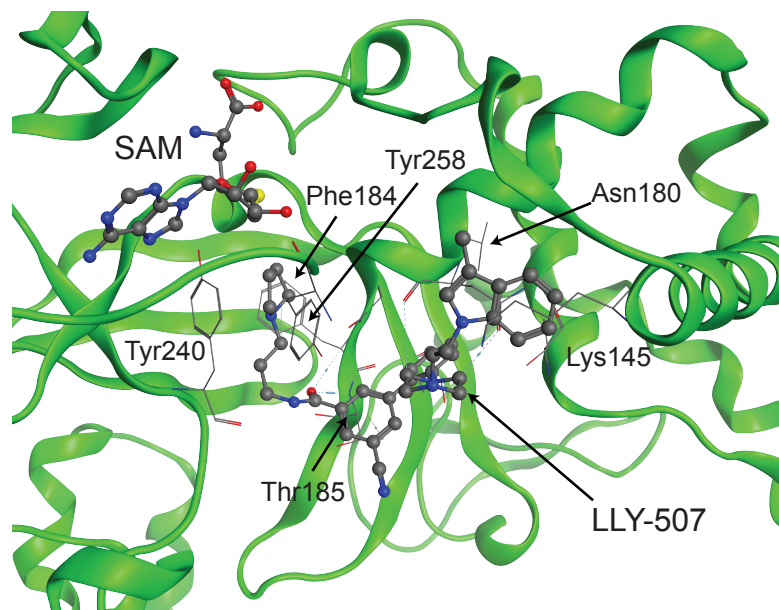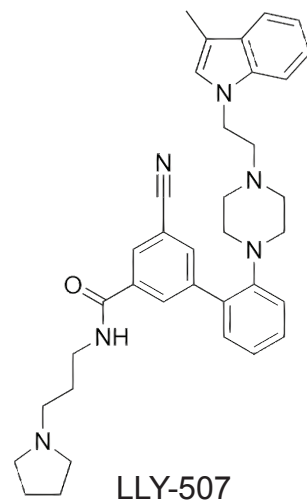

B)

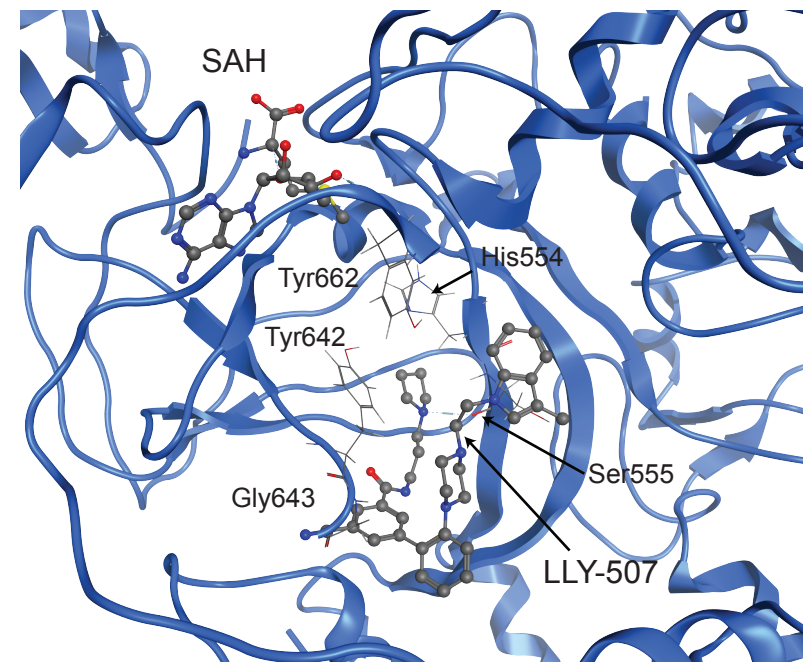

C)

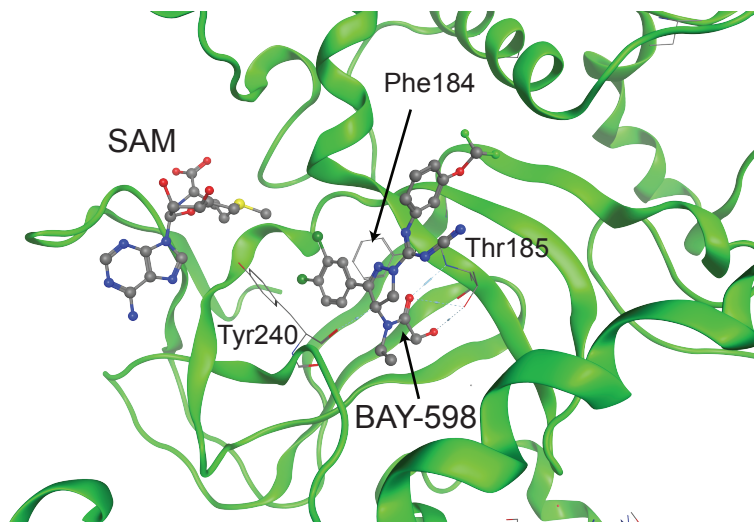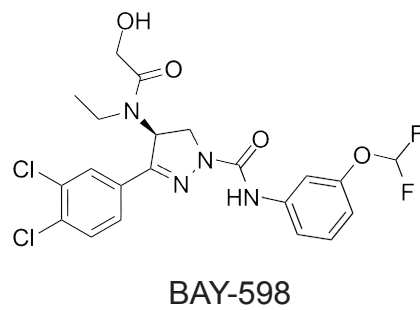

D)

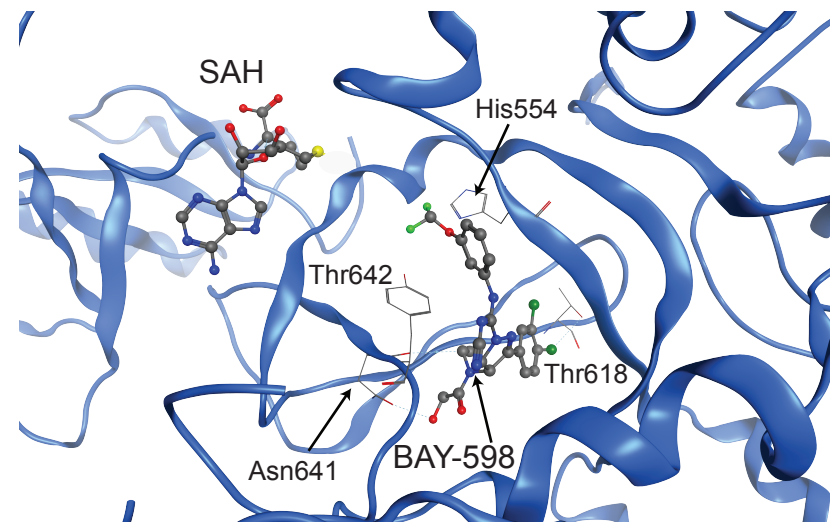

Supplement: S4 Fig — Views of the co-crystal structure of LLY-507 with HsSMYD2 (PDB ID: 4WUY; Panel A) compared to the predicted binding of LLY-507 with the homology model of Smp_000700 (Panel B). Similar comparisons were made between the co-crystal structure of BAY-598 with HsSMYD2 (PDB ID: 5ARG; Panel C) and the homology model of Smp_000700 (Panel D). SAM (S-adenosyl methionine, for HsSMYD2), SAH (S-adenosyl homocysteine, for Smp_000700) and the compound structures are shown as ball-and-stick diagrams, coloured by atom type: grey for carbons, red for oxygen, blue for nitrogen. The human and parasite proteins are shown as green and blue ribbon, respectively. Residues interacting with the compounds are shown in stick mode and the relative numeration refers to their positions on the full-length protein sequence. For clarity, hydrogens, small portion of the ribbon and protein side chains and backbones (except for the highlighted residues) are not shown. (PDF) [file pntd.0007693.s005.pdf]

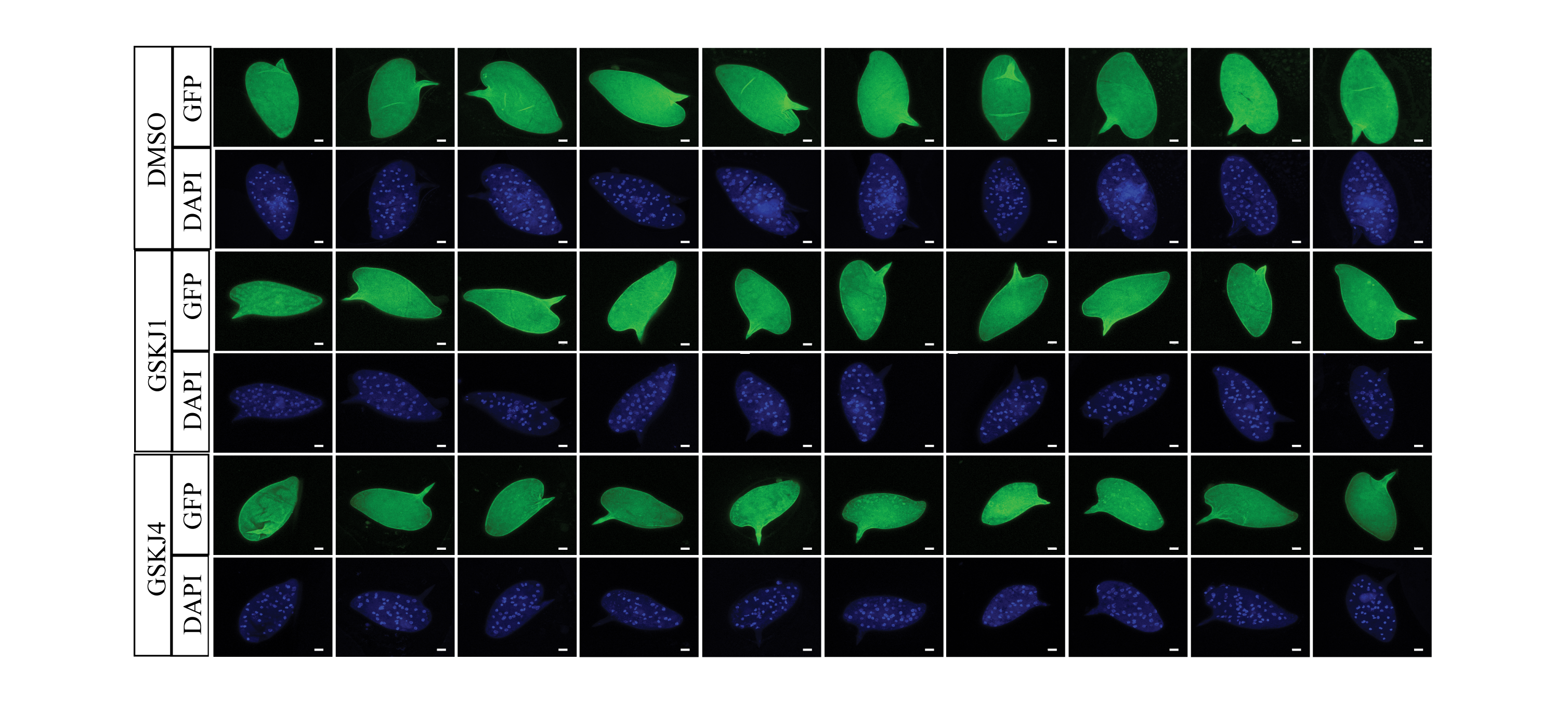

Supplement: S5 Fig — Representative IVLE phenotypes (GFP = eggshell surface autofluorescence; Ex = 488 nm, Em = 519 nm and DAPI = vitellocytes; Ex = 405 nm, Em = 458 nm) from schistosome pairs co-cultivated in GSK-J4 (0.2 μM), GSK-J1 (6.25 μM) or 0.625% DMSO for 72 h. (TIF) [file pntd.0007693.s006.tif]
