# Supplementary material for: The repositioning of epigenetic probes/inhibitors identifies new anti-schistosomal lead compounds and chemotherapeutic targets
Source: PLoS Negl Trop Dis. 2019 Nov 15;13(11):e0007693. doi: 10.1371/journal.pntd.0007693 (PMC6881072; doi:10.1371/journal.pntd.0007693)
Supplement: S3 Fig — (A) Ramachandran plot showing the dihedral Psi and Phi angles of amino acid residues within the catalytic domain of Smp_000700 (SET domain, 413 aa in length). This analysis illustrates that 98.6% of modelled residues satisfy stereochemical parameters. In fact, various residues lie in the general favoured regions (black symbols in blue and orange areas on the graph) and the allowed regions (orange symbols in blue and orange areas on the graph). Very few residues lie within the white field, which represents disallowed regions. (B) Z-score of Smp_000700’s SET domain provided by ProSA-web. The black dot (highlighted by the arrow) represents this Z-score (-7.11) in relation to all protein chains in PDB determined by X-ray crystallography (light blue area) or NMR spectroscopy (dark blue area) with respect to their length (x-axis representing the protein length in terms of number of residues). Our model is located within the space occupied by protein structures solved by X-ray crystallography. (C) Smp_000700 model quality (over SET domain) assessed by the protein verification tool ERRAT. Error values are plotted as a function of a sliding 9-residue window; poorly supported model residues (highest bars on the Errat Plot) are coloured red (rejected at 99% confidence level or above) or yellow (between 95% and 99% confidence levels). Regions of the structure not rejected are shown in green. Overall ERRAT score of Smp_000700’s SET domain is 88.15%. (D) Evaluation of Smp_000700 homology model (SET domain) was additionally conducted by Verify3D, which determines the compatibility of an atomic tertiary model (3D) from its own primary amino acid sequence (1D). As a result, 81.90% of the SET domain residues have a good score (> = 0.2) compatible with the formation of a stable 3D structure. (E) Quality structure assessment summary of Smp_000700 homology model (SET domain) and the corresponding human template (SMYD3, PDB ID: 5EX3). This final table summarises the results of the struct [file pntd.0007693.s004.pdf]

A)

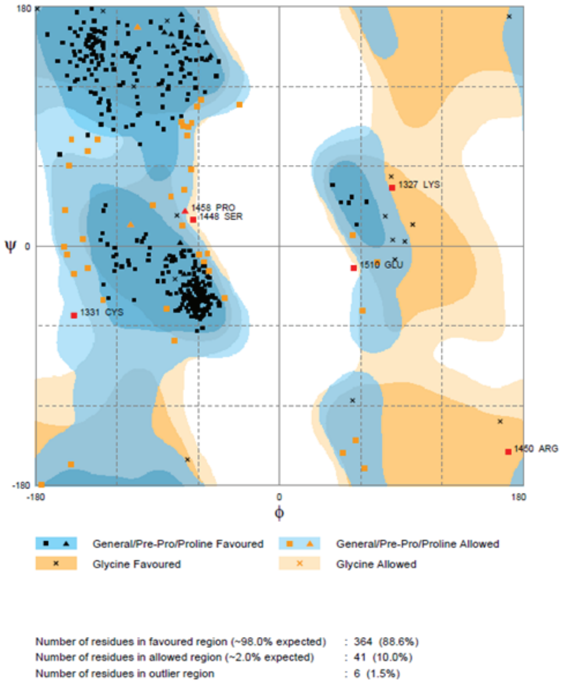

B)

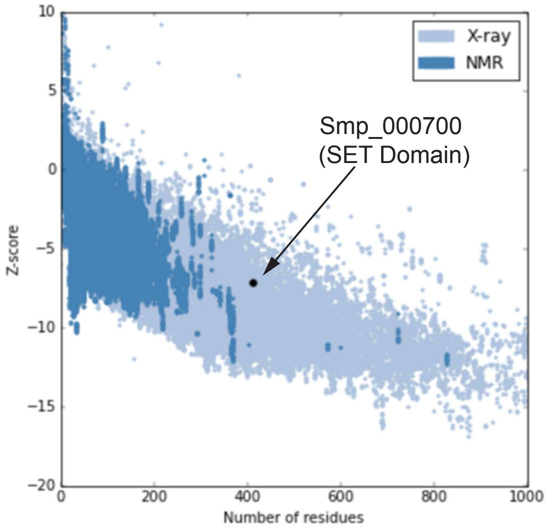

C)

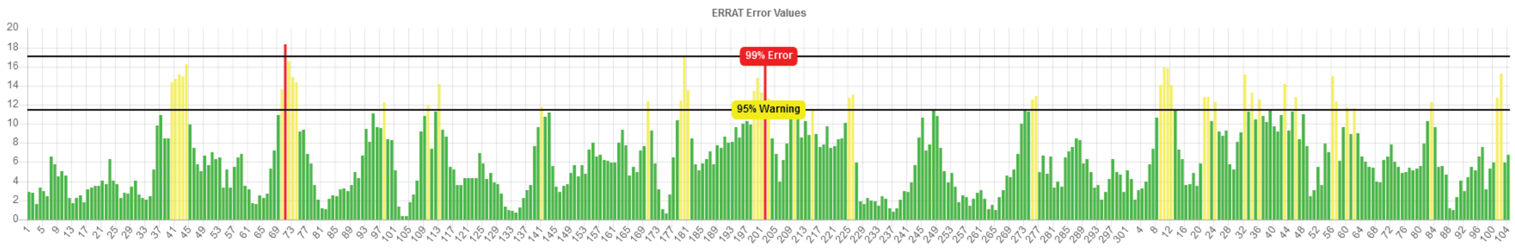

D)

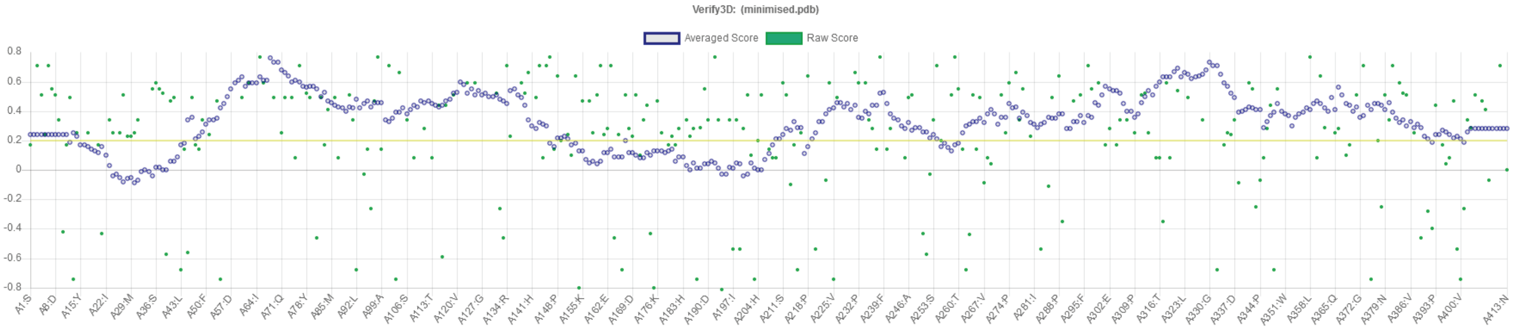

E)

		Smp_000700	Human template (5EX3)
RAMPAGE	Number of residues in favoured region (~98.0% expected)	88.60%	97.90%
	Number of residues in allowed region (~2.0% expected)	10%	2.10%
	Number of residues in outlier region	1.50%	0
ProSA-web	Z-score	-7.48	-9.8
ERRAT	Good high resolution structures around 95% or higher. For lower resolutions (2.5 to 3A) the average overall quality factor is around 91%	88.15%	95.93%
Verify 3D	80% of the amino acids have scored >= 0.2	81.90%	99.07%
