# Supplementary material for: The repositioning of epigenetic probes/inhibitors identifies new anti-schistosomal lead compounds and chemotherapeutic targets
Source: PLoS Negl Trop Dis. 2019 Nov 15;13(11):e0007693. doi: 10.1371/journal.pntd.0007693 (PMC6881072; doi:10.1371/journal.pntd.0007693)
Supplement: Attachment — Submitted filename: Response to reviewers_Whatley and Padalino.docx [file pntd.0007693.s007.docx]

**Point-by-point Response to the Reviewers’ Comments on PLoS NTDs (PNTD-D-19-01335)**

**We thank the three reviewers very much for their detailed and constructive comments on our manuscript entitled: “The repositioning of epigenetic probes/inhibitors identifies new anti-schistosomal lead compounds and chemotherapeutic targets”, submitted to *PLoS NTDs*. Below, we provide a point-point-response (in blue) to the Reviewers’ comments (in black).**

Reviewer Comments:

Reviewer #2: Response to clarification of minor queries regarding methodology.

What strain of Mus musculus? (Line 149)

As stated in the Materials and Methods section, the strain of *Mus musculus* used in this study was Tuck Ordinary (TO).

Schistosomula were cultured with compound at 37 degrees – were they also imaged at 37 or at room temperature? (Line 188)

Schistosomula were imaged within an environmentally controlled high content imaging platform (Molecular Devices, ImageXpress Micro XL microscope) at 37°C with 5% CO_2_ in a humidified atmosphere during the screening process. This information has now been added to the manuscript.

Reference 50 (Paveley et al., 2012) is mentioned regarding phenotype and motility. However, some details on this package could be clarified. Is this package publicly available? (Line 189)

The image analysis pipeline published by Paveley *et al.,* (2012) was shared by Professor Quentin Bickle and his research group to allow us to build a similar platform at Aberystwyth University, which mirrored the capabilities of the platform at the London School of Hygiene and Tropical Medicine. As such, the image analysis pipeline is not ours to make public. Additionally, the software required to run the pipeline (Pipeline Pilot (v8.5)) and image the schistosomula (Meta Xpress, Molecular Devices, UK) are both products for which licenses have to be purchased. Nevertheless, as mentioned above, a detailed description of how these software packages cooperatively work to facilitate schistosomula screening has already been published (Paveley *et al.,* 2012).

Reference 50 (Paveley et al., 2012) is mentioned regarding phenotype and motility. What was the rational for the hit thresholds (dotted line figure 1A and 1B)? (Line 189)

The development and rational for the hit thresholds (-0.35 for motility, and -0.15 for phenotype) are described fully in Paveley *et al.,* (2012) (our reference 50). To summarise Paveley *et al.,* (2012), the hit thresholds of -0.35 for motility and -0.15 for phenotype were defined following the screening of three individual 384-well plates with 8 different treatments (media, media + DMSO at 0.625%, Praziquantel, Oltipraz, Oxamniquine, methylclonazepam, Ro15-5458, and dihydroartemisinin all tested in 0.625% DMSO) at 10 µg/ml. Their finding was that thresholds of -0.35 and -0.15 accurately defined whether a treatment well was a ‘hit’ (drugs/compounds used at 10 µg/ml and subsequently validated at 10 µM, where ≥70% of the schistosomula was damaged or dead) or a ‘non hit’ (media or media + DMSO). As these hit thresholds were thoroughly reported in a previous publication, cited here, we have purposely not repeated this information in the current study. We have directed the reader to the original manuscript describing, in detail, the methodology originally developed and employed in our study (as well as several others previously published by us).

Suggest change to Table 2- state how adult worms have been screened and pop in text line 409

We have now summarised (from the Materials and Methods section) a description of how both schistosomula and adult worms were cultured as well as how EC_50_s were calculated. This is now included in footnote section of Table 2.

For RNAi, what concentration of siRNA duplexes were used?

As cited in the Materials and Methods (references 20 and 21), we used a siRNA duplex concentration of 50 ng/μl for initiating adult worm siRNA. The manuscript has now been amended to include this information.

How many worms sample input are used in this assay? (line 263)

To confirm, we utilised 21 male and 21 female worms (or 21 worm pairs) for each condition (luc or smp_000700 siRNAis). The manuscript has now been amended to include this information.

The structures of the chemicals shown in table 2 should be included. It may not be convenient to include them in the actual table 2, but perhaps a supplemental table or in text form (for example, in SMILES format).

This information (structures and SMILES as well as a variety of other compound-associated metrics) was already included in S1 Table for all SGC compounds screened in this study.

While this is not essential, I am interested in whether pharmacological or genetic inhibition of histone methylation effects mitotic activity of somatic neoblasts or germ tissues (testies, ovaries, vitellaria)? Is this the mechanism by which egg laying is arrested?

We have not directly assessed this in the current study (e.g. measuring the level of EdU incorporation in drug- or RNAi- treated schistosomes). However, as it has been previously shown that both A366 (G9a/GLP HMT inhibitor) and GSK343 (EZH1/H2 HMT inhibitor) completely block miracidia to sporocyst transformation (Roquis *et al.,* 2018), histone methylation may indeed affect the mitotic activity of somatic neoblasts (and germ cells). This is an area of current investigation in the laboratory.

The conclusions are well supported by the data. Some discussion could elaborate the repurposing of epigenetic probes / inhibitor compounds. As mentioned in the introduction, praziquantel is not effective against juvenile life stages (~4 week, liver stage immature worms). Granted, ex vivo assays on these worms are more challenging – but is anything known about the expression of targets such as Smp_007000 across the schistosome life cycle? Given the effects on egg laying, it would be interesting whether these are differentially expressed in males and females or in the gonads (PMID: 27499125).

In the conclusions, we have indicated that repurposing of these compounds as schistosomiasis control agents could be rapid. We purposely did not want to expand upon this further due to this study’s data originating from *in vitro* assays only. Nevertheless, our introduction provides context as to why a repositioning approach for NTDs (e.g. schistosomiasis) can benefit global health. The expression of *smp_000700* has previously been reported by us (Padalino *et al.,* 2018; ref 34, Figure 2A) across the *S. mansoni* lifecycle and illustrates that both 3 wk and 5 wk worms express this transcript. Furthermore, the same study reports that *smp_000700* is more abundant in adult males compared to adult females. We have now included a sentence indicating the gonadal expression (Lu *et al*., 2017) of this transcript (ovary > testes) to provide further context for the egg production deficiency observed upon functional genomics manipulation of *smp_000700*.

Reviewer #1:

I list below several minor points to be corrected but the only major point to be addressed is why authors used such a small incubation time (20h) with cytotoxicity assays on the human cell line for calculating selectivity indexes. It's an issue since parasite assays were all performed with 72h incubation time. The authors should generate new data with 72h incubation or make a strong case justifying their choice for 20h since keeping the later should overestimated the selectivity/safety of compounds.

Our rationale for choosing a 20 h (effectively overnight) window for HepG2 cytotoxicity estimations is based on two reasons. The first reason originates from the literature (Hsieh et al., 2017. <https://doi.org/10.1371/journal.pone.0177902)>. Here, as part of the U.S. Tox21 screening program, HepG2 cytotoxicity of 10K chemicals was investigated at 0, 8, 16, 24, 32, and 40 h of exposure in a concentration dependent fashion. The results of these screens indicated that maximal HepG2 cytotoxicity (measuring metabolic activity, which is how we have measured cytotoxicity in our study) of active compounds was found before 24 hours in 91% of the assays. This large-scale cytotoxicity screen indicated to us that an appropriate window for testing the, already well-characterised, repositioned SGC epigenetic probes/inhibitors on a uniform monolayer of HepG2 cells was 20-24 (O/N) h. We have now included this reference in the Materials and Methods to provide further justification of the 20 h interval. The second reason relates to the nature of the biological material we are comparatively testing in our *in vitro* assays. In comparison to a monolayer of HepG2 cells, schistosomes (schistosomula and adult worms) are much larger, multi-cellular, multi-tissue organisms covered by a heptalaminate membrane. We reasoned that compound-induced anthelmintic activities would take longer to develop in these metazoan organisms when compared to the HepG2 cells. This is the rationale for quantifying anti-schistosome phenotype and motility metrics at 72 h. Indeed, this can be seen for PZQ treated schistosomula (Figure 1, below). At 24 and 48 h, the PZQ-treated schistosomula are not considered a hit in the Roboworm assays, where at 72 h, they are considered a hit (within the intersection of both motility and phenotype axes).

**Figure 1: The anti-schistosomula activity of praziquantel (10 µM) at 24, 48 and 72 h.** Three separate plates were set up with a total of 4 wells of PZQ alongside 8 wells of Auranofin (positive control) and 16 wells of DMSO (negative control) on each plate. The average of all DMSO and Auranofin (AUR) wells across all three plates were taken and a single average value for both treatments was plotted. Each of the individual PZQ readings for each time point was plotted. At 24 h, PZQ has no effect on either motility or phenotype of the schistosomula. However, with increased incubation time, PZQ begins to reduce motility and negatively affect phenotype. At 72 h, PZQ induces motility and phenotype metrics that fall within the hit zone (within the quadrant occupied by AUR). The Z’ for each of the 3 plates were as follows: 24 h – Phenotype 0.58541 Motility 0.61772, 48 h - Phenotype 0.50272 Motility 0.41385, 72 h Phenotype 0.38347 Motility 0.49464.

These examples illustrate why we have chosen to examine the effects of the SGC compounds at different time-points on HepG2 cells (20 h) and schistosome parasites (72 h). Nevertheless, we are only using HepG2 cytotoxicity at 20 hr as a red flag and not a go/no-go decision point (now explicitly stated in the revision). We believe that the true value of these screens, using the epigenetic probes/epigenetic inhibitors, is to identify putative druggable targets with *S. mansoni*. It is clear that the therapeutic potential of the tested drugs (developed against human targets), as they currently exist, may be limited unless medicinal chemistry is applied to develop more selective compounds. These types of investigation are currently under study in our laboratory and will be reported as soon as they have been completed.

Minor points:
Methods:

1: Compound acquisition, storage and handling: Please indicate where appropriate that compounds structures can be found at Table S1.

We have now included where compound structures can be found in Table 1 and Figure 1 legends.

2: Homology modelling and epi-drug docking:

2a: Provide (as supplementary material) evidence for model validation and quality, having special attention to any issues involved in the proposed docking site.

We have now included the requested information as part of the supplementary files (S3 Figure). The catalytic SET domain (413 aa) was evaluated by four different tools (Ramachandran plot analysis, ProSA-web, ERRAT and Verify3D), which confirmed a good quality of the proposed model without any issue over the substrate binding pocket (docking site of LLY-507 and BAY-598).

2b - Are all the EP/Eis know to be competitive with the substrates? if so, authors should state it earlier.

Not all the EP/EIs are competitive inhibitors of the substrates; for example, SGC0946 binds to the cofactor site of DOT1L. We have now included a column in S1 Table that describes mechanism of action of each of the SGC compounds.

A general comment: the reality of therapy for schistosomiasis is that compounds need to be active after a single dose, where active means worm killing. Blocking motility or egg production after extended exposure to a compound will not be adequate for novel therapies. Did the compounds tested actually _kill_ the worms?

The ‘hit’ threshold for schistosomula describes a condition where ≥70% of schistosomula within a treatment well were dead or damaged (Paveley *et al.,* 2012). In screening of adult worms, as described by Ramirez *et al.,* (2009), worms were described as dead (score of 0) if no motility or gut peristalsis could be observed under a stage microscope at 40X magnification. These are the metrics we (and others) have used during our *in vitro* search for anti-schistosomal compounds. Without further assessing for viability (using viability dyes) or washing out compounds and measuring recovery (difficult to do using a high-throughput platform), it is impossible to tell if the metrics used in this study are quantifying immotile/affected parasites or dead parasites. However, for compounds being progressed as potential treatments for schistosomiasis, we agree with this reviewer that viability in addition to many other pre-clinical factors should be considered. The intention of our study was to highlight repositioned epigenetic probes/epigenetic inhibitors (and their putative schistosome targets) as novel schistosomiasis leads. Clearly, follow-on experiments would need to be developed to address other pre-clinical characteristics of these compounds.

- Results:

Lines 311-313: Kd, Ki or IC50? the term potency may be misleading since it's usually expressed as an inverse proportional term. Then, higher potency may actually mean < 100nM.

While the values included in this sentence are derived from the SGC’s differential definition of epigenetic probes and epigenetic inhibitors - Please see Scheer et al., 2019, *Nature Communications*, doi.org/10.1038/s41467-018-07905-4, we agree with the reviewer that the word ‘potency’ may be misleading. Therefore, we have replaced this word with activity.

Lines 348-349: Figure 1 legend: Please indicate if dashes crossing vertical lines connecting each replicate mean averages or medians.

Each bar represents average phenotype or motility scores derived from the 2-3 replicates. We have now included this statement in the Figure 1 legend.

Line 385: Please, complement S1 figure by including chemical structures for the compounds.

All compound structures were already included in S1 Table.

Line 386-387: authors contend the fact that none of these chemotype controls demonstrated anti-schistosomula activity suggests that specificity of the hits for the S. mansoni homologue is similar to that found for the original human target but other interpretation would be they lack proper SAR for these targets. It should be discussed.

We have added in a line that addresses this reviewer’s comment:

‘Further SAR of the chemotype-matched controls and their related hit EPs/EIs with the schistosome target or whole organism could help provide evidence to support this contention.’

Table 2: All EC50 and CC50 should be accompanied of standard errors or 95% confidence intervals.
Also indicate incubation time below headings. In the table footnote, please define "accurate".

Where possible, we have now included 95% confidence intervals (CIs) for all EC_50_s of adult worm assays and CC_50_s of HepG2 assays. In all instances, these values are approximates and labeled as such in the Table 2 footnote. For schistosomula, we cannot calculate 95% CIs as the dose response titrations (to generate EC_50_ estimates) were only performed once (i.e. each concentration of compound was tested against one well of ~ 100 - 120 schistosomula). We are indicating these tests with an ‘NC’ (not calculable) in the Table 2 footnote. This is in contrast to the single point (10 µM) assays reported in Figure 1, where anti-schistosomula tests were repeated 2 or 3 times. In order to calculate an accurate 95% CI for anti-schistosomula EC_50_s, greater numbers of titration points (greater than the five concentrations used here) and repeat analyses (multiple wells) are required. As we only wanted to cross-compare the anti-schistosomula activities of this collection of compounds for further triaging, we believed a single titration experiment was justifiable in our decision process. Nevertheless, the statistical method used to indicate that our dose response titrations of schistosomula were within a well-recognized window of assay acceptability was the Z´ factor. In all dose response titrations, Zˊ values obtained from these screens were 0.44 for phenotype and 0.41 for motility. This statistically-derived QC metric of high-throughput assays is now also included in the Table 2 footnote.

For adult worm dose response titrations, EC_50_ approximates were derived from the repeat assays (i.e. repeat titrations at 50 µM, 25 µM, 12.5 µM and 6.25 µM). Unfortunately, for some of these compounds, the motility score range across the concentrations tested was too narrow (WHO-TDR scores of 3 or 4 across all concentrations) and, therefore, an accurate 95% CI could not be calculated (NC). For other compounds, the range was too wide to calculate accurate 95% CI (also indicated by NC). Without testing additional concentrations of compounds, an accurate EC_50_ cannot be determined and a 95% CI cannot be reported. Similar to the schistosomula assays, we only wanted to use these EC_50_ estimates to further triage the compounds for further mechanistic studies (in this case BAY-598, LLY-507 and GSK-J4). We felt that the additional use of mice to generate more adult worms for further titration points was not justifiable (against the 3R principles) in this instance.

For the CC_50_ measurements, the 95% CI was generated from a minimum of 3 biological dose response repeats (100 µM, 10 µM, 1 µM, 0,1 µM and 0.01 µM) and, where possible, values were reported. For those compounds not demonstrating cytotoxicity even at the highest concentrations tested, we were not able to determine EC50s or 95% CIs as explained in the Table 2 footnote.

We have additionally indicated the incubation times in the footnote. The term ‘accurate’ was meant to indicate that neither an EC_50_ or CC_50_ could be determined for those highlighted compounds due to the number of titration points included. Accurate values required increased numbers of titration points (e.g. > 100 µM for the cell cytotoxicity screens). Therefore, we have removed the word ‘accurate’ from the table’s footnote as it may have caused confusion.

Lines 413-415: are there any SAR to be discussed for compounds LLY-507 and BAY-598?

We have thoroughly discussed how the structures of both LLY-507 and BAY-598 could affect the binding to Smp_000700 in the manuscript. We have now provided some further text related to SAR:

‘The variation in predicted binding modes of LLY-507 and BAY-598 to Smp_000700 (i.e. LLY-507 adopting a confirmation analogous to the human homolog; BAY-598 binding to an adjacent hydrophobic pocket) may explain the difference in anti-schistosomal activity observed (LLY-507 > BAY-598). Further SAR could be helpful in providing answers to the differential activity of these two putative Smp_000700 interactors.’

Line 444: "strong" for "close"

Done.

Line 463-478: authors should make their conclusions lighter using expressions like "is predicted to bind..." or "predicted interaction may prevent..."

We have tempered this text in line with the reviewer’s comments.

Line 523: Please clarify why GSK-J4 is a prodrug. How is the drug processed?

According to the SGC website and associated documentation (e.g. Kruidenier et al., *Nature* volume 488, pages 404–408 (2012)), GSK-J4 is cell permeable prodrug rapidly hydrolysed by intra-cellular esterases to GSK-J1, a potent selective jumonji H3K27 demethylase inhibitor. We have now included reference to GSK-J4 processing in the manuscript.

Typos:
Line 71: theat for threat

Done

Line 127: "cultured in this" for "cultured with this"

Done

Line 452: "β stands" for "β strands"

Done

We would like to thank the three Reviewers very much for their detailed and very constructive comments, which have helped us to improve our manuscript. We hope that this point-by-point response could clarify the open questions and that our suggested revisions are clear and convincing. We are, of course, happy to discuss any further open issues.

Kind regards,

Karl F Hoffmann (on behalf of the authors)
